# Respondent characteristics associated with adherence in a general population ecological momentary assessment study

**DOI:** 10.1002/mpr.1972

**Published:** 2023-05-15

**Authors:** Aja Murray, Yi Yang, Xinxin Zhu, Lydia Speyer, Ruth Brown, Manuel Eisner, Denis Ribeaud

**Affiliations:** ^1^ Department of Psychology University of Edinburgh Edinburgh UK; ^2^ Clinical Psychology Department University of Edinburgh Edinburgh UK; ^3^ Institute of Criminology University of Cambridge Cambridge UK; ^4^ Jacobs Center for Productive Youth Development University of Zurich Zurich Switzerland

**Keywords:** adherence, ecological momentary assessment, experience sampling, missingness, non‐response

## Abstract

**Objectives:**

Ecological momentary assessment (EMA) has seen an explosion in popularity in recent years; however, an improved understanding of how to minimise (selective) non‐adherence is needed.

**Methods:**

We examined a range of respondent characteristics predictors of adherence (defined as the number of EMA surveys completed) in the D2M EMA study. Participants were a sample of *n* = 255 individuals drawn from the longitudinal z‐proso cohort who completed up to 4 EMA surveys per day for a period of 2 weeks.

**Results:**

In unadjusted analyses, lower moral shame, lower self‐control, lower levels of self‐injury, and higher levels of aggression, tobacco use, psychopathy, and delinquency were associated with lower adherence. In fully adjusted analyses with predictors selected using lasso, only alcohol use was related to adherence: beer and alcopops to higher adherence and spirits to lower adherence.

**Conclusions:**

These findings provide potential insights into some of the psychological mechanisms that may underlie adherence in EMA. They also point to respondent characteristics for which additional or tailored efforts may be needed to promote adherence.

## 1 INTRODUCTION

Ecological momentary assessment (EMA) methodologies are data collection designs that capture participants' moment‐to‐moment experiences in the flow of their daily lives, often using brief smartphone‐based surveys completed multiple times a day. EMA has seen an explosion in popularity in the study of mental health and psychiatric disorders (Russell & Gajos, 2020). The method is thought to be particularly valuable in younger populations where smartphone ownership levels are high and their use is well‐embedded within daily routines.

Poor adherence in EMA studies (defined in terms of the number of the prompts delivered to participants' smartphones that are completed) has important consequences for the quality of the inferences that can be made. It reduces the available measurement observations (and correspondingly statistical power) and may induce biases when adherence is not random with respect to the constructs of interest in a study (Ottenstein & Werner, 2021). Knowledge of the respondent characteristics that influence adherence can help mitigate this loss of observations and associated bias. For example, where certain respondent characteristics (e.g., depression) are shown to be associated with lower adherence, mitigation strategies can be designed, such as baseline oversampling of respondents with these characteristics, or providing tailored protocols to improve their adherence. Similarly, it can flag respondent characteristics that may be valuable to measure to indicate participants at risk of poor adherence for tailored engagement strategies (‘adaptive designs’) or to use in statistical adjustments (e.g., in weights, multiple imputation, or as auxiliary variables) to reduce biases.

Despite the potential value of knowledge of the respondent characteristics that predict EMA adherence, research on the topic has been limited. It is particularly important to identify characteristics that past research or theory suggests could be related to both adherence and the variables of interest in a study. Selection on these characteristics not only reduces the available sample size but can also induce bias in parameter estimates. For example, poorer adherence of individuals with higher levels of depression could distort the associations between depressive symptoms and candidate predictors or outcomes (e.g., sleep, emotion regulation, suicidal ideation) being investigated in an EMA study.

In the broader attrition literature, there is evidence that sociodemographic variables may be related to mental health such as gender, educational level, and marital or family status being associated with non‐response (Watson & Wooden, 2009). Consistent with this, some studies have identified sociodemographic predictors, including age and male gender as predictors of EMA non‐adherence in studies examining outcomes such as mental health and substance use (Martinez et al., 2021; Messiah et al., 2011; Ono et al., 2019; Silvia et al., 2013; Sokolovsky et al., 2014; Turner et al., 2017). However, these effects have not always been replicated (Courvoisier et al., 2012; Hartley et al., 2014; Williams‐Kerver et al., 2021). There is also a lack of research on the effects of sociodemographic characteristics on EMA adherence in general population samples, despite interest in illuminating moment‐to‐moment influences on and manifestations of mental health symptoms in both clinical and non‐clinical populations.

In psychiatric research, understanding the effects of mental health symptoms on adherence is particularly critical (Rintala et al., 2019). Symptoms and sequelae of mental health conditions, such as a lack of energy and motivation have strong face validity as factors that could increase difficulty of EMA adherence; however, the empirical evidence on this is inconsistent. Gershon et al. (2019) found that young people with bipolar disorder showed lower EMA adherence than controls. Similarly, Silvia et al. (2013) found that adherence was lower for individuals who were high in positive schizotypy, depressive symptoms, and hypomania. Another study found that participants with affective disorders experienced a higher perceived burden compared to those with remitted or unaffected status; however, they nonetheless showed high adherence on average and their adherence levels did not differ significantly from other groups (van Genugten et al., 2020). Rintala et al. (2019) found no relation between depressive symptoms and adherence; however, they found that individuals with psychosis showed lower adherence than healthy individuals.

Evidence does, however, support the idea that substance use/abuse could exacerbate adherence difficulties in EMA (Jones et al., 2019). Comparisons both between and within studies support the idea that substance use is associated with lower adherence (Jones et al., 2019). For example, in a study of adolescent smokers, Sokolovsky et al. (2014) found that higher rates of smoking and alcohol use predicted lower EMA adherence. Similarly, Messiah et al. (2011) found, in a sample of undergraduate university students, that being a polysubstance user was associated with lower adherence.

While the above studies have provided insights into some key respondent characteristics that impact EMA adherence, a comprehensive understanding of potential predictors is lacking. In particular, previous research has focused on a limited range of domains of psychiatric symptoms leaving other plausible predictors of adherence under‐explored. Symptoms associated with attention deficit hyperactivity disorder (ADHD), for example, have strong potential as predictors of adherence whilst also being important outcomes in psychiatric research. ADHD symptoms are associated with daily life difficulties in planning and organisation, with maintaining focused concentration, and with impulsivity (American Psychiatric Association, 2013), all of which could translate into difficulties with sustained engagement with EMA protocols in research contexts. Indeed, previous research has found ADHD symptoms to be associated with drop‐out from longitudinal studies (Eisner et al., 2018) and with lower levels of participation willingness in EMA (Murray, Ushakova, et al., 2022).

Likewise, symptoms related to antisocial behaviour disorders (e.g., conduct disorder and antisocial personality disorder) such as a lack of prosocial emotions/motivations and non‐compliance with societal norms (APA, 2013) are plausible predictors of EMA adherence. Indeed, previous research has suggested that higher levels of antisocial behaviour/traits are associated with greater drop‐out rates in longitudinal studies (Brame & Piquero, 2003) whilst prosocial traits may be related to greater willingness to participate in research (Critchley et al., 2012). Similarly, prosocial motivations are often identified as important in driving research participation (Del Savio et al., 2017).

Finally, previous research has focused primarily on symptoms of disorders that correspond to psychiatric diagnoses. However, reflecting a broader conception of mental health as including transdiagnostic features and encompassing not only the lack of mental illness, but also the presence of psychosocial strengths (Fusar‐Poli et al., 2020), a diverse range of outcomes beyond psychiatric disorder symptoms are often of interest in mental health research. These broader psychosocial wellbeing outcomes may also be related to EMA adherence. For example, characteristics such as generalised trust, self‐efficacy or self‐control, may promote sustained commitment to and engagement with EMA protocols, whereas issues such as perceived stress or social exclusion may be associated with greater adherence challenges.

### 1.1 The current study

Given the importance of illuminating the predictors of EMA adherence—especially predictors related to mental health itself—in mental health research, the current study thus aims to provide a more comprehensive exploration of the sociodemographic, mental health, and broader psychosocial well‐being factors that may be related to non‐response within EMA paradigms. We use data from the D2M study (Murray, Speyer, et al., 2022) which has a relatively standard EMA design (Wrzus & Neubauer, 2023), sampling young people (aged 21 at the time of data collection) from the general population and administering smartphone‐based EMA surveys four times per day over a 14‐day period. We leverage the fact that participants in this EMA study also completed comprehensive survey measures prior to their participation that can be used to predict their adherence to the EMA protocol. Taking an exploratory approach, we include a wide range of candidate predictors of adherence but with a particular focus on those that may illuminate the psychological mechanisms of EMA adherence and/or which result in biased psychiatric and mental health EMA studies.

## 2 METHODS

### 2.1 Participants

Participants were *n* = 255 young adults from the ‘Decades‐to‐minutes’ (D2M) sub‐study (Murray et al., 2020) of the longitudinal Zurich Project on Social Development from Childhood to Adulthood (z‐proso; Ribeaud et al., 2022). D2M is an EMA sub‐study that aims to illuminate the long‐term developmental and daily life proximal influences on mental health and behaviour problems (Murray, Speyer, et al., 2022). Z‐proso began in 2004 when participants were aged 7 and used a stratified random sampling procedure to select schools from Zurich, Switzerland. All children within selected schools were invited to participate. Previous analyses have suggested that the z‐proso cohort is generally representative of the underlying same‐aged population in Zurich, which is ethnically and socio‐economically diverse (Ribeaud et al., 2022). Further, analyses of participation and attrition have suggested that there has been minimal non‐random participation and attrition in the study (Eisner et al., 2018).

The D2M sub‐sample was a convenience sample from the age 20 wave of z‐proso (with an average age of 21 at the time of EMA data collection). The sub‐sample is 61% female with a mean childhood household International Socio‐economic Index of Occupational Status (ISEI) occupational prestige score of 49 (Ganzeboom et al., 1992). Though a majority of the participants (*n* = 159) had a primary caregiver born in Switzerland, primary caregivers came from a diverse set of nations. The most common among these include Sri Lanka (*n* = 18), Portugal (*n* = 10), Serbia & Montenegro (*n* = 9), Germany (*n* = 7), Turkey (*n* = 6), and the Dominican Republic (*n* = 4). Further descriptive characteristics of the sample are provided in Table 1, including migration status and levels of mental health and psychosocial wellbeing.

**TABLE 1:** Descriptive statistics for predictors.

	*N*	Mean	*SD*	Median	Min	Max
Gender	98 male, 156 female					
Migration status	At least one parent born in Switzerland = 157					
	Both parents born abroad = 92					
ISEI	236	50.538	19.702	52.000	16.000	90.000
ADHD	252	2.871	0.672	2.778	1.444	4.889
Depression symptoms	254	2.300	0.810	2.111	1.000	4.667
Anxiety symptoms	254	2.507	0.882	2.500	1.000	5.000
Psychosis symptoms	254	1.444	0.466	1.333	1.000	3.167
Aggression	254	1.430	0.297	1.368	1.000	2.737
Prosociality	252	3.874	0.523	3.900	1.200	5.000
Tobacco use	253	3.316	1.865	3.000	1.000	6.000
Alcohol use (beer/wine/alcopops)	253	3.854	1.336	4.000	1.000	6.000
Alcohol use (spirits)	253	3.387	1.247	4.000	1.000	6.000
Cannabis use	253	2.427	1.643	2.000	1.000	6.000
Self‐injury	254	1.134	0.442	1.000	1.000	4.000
Delinquency	253	0.123	0.114	0.143	0.000	0.571
Psychopathy	254	1.709	0.477	1.667	1.000	3.667
Low self‐control	254	2.025	0.390	2.000	1.200	3.300
Moral shame	254	2.965	0.647	3.000	1.000	4.000
Self‐efficacy	254	2.822	0.451	2.800	1.600	4.000
Social exclusion	254	1.490	0.556	1.333	1.000	3.500
Stress	254	2.958	0.889	2.750	1.000	5.000
General trust	254	2.409	0.670	2.333	1.000	4.000

Abbreviations: ADHD, attention deficit hyperactivity disorder; ISEI, International Socio‐economic Index of Occupational Status.

Previous analyses have explicitly compared D2M to the broader z‐proso cohort and suggested that the D2M sub‐sample is slightly higher in socioeconomic status (SES) and lower in aggression but higher in stress (Murray, Speyer, et al., 2022). The sub‐sample does not, however, appear to differ from the main z‐proso sample on a range of other characteristics, including ADHD symptom levels, internalising problems, and substance use (Murray, Speyer, et al., 2022).

### 2.2 Data collection procedure

Participants were recruited at the age 20 main data collection wave of z‐proso in 2018 and those who agreed to participate were followed up and provided with information on how to participate in an EMA sub‐study. The study was delivered via an application provided by *LifeDataCorp LLC* downloaded to participant smartphones. It involved completing brief surveys 4 times a day over a 2‐week period. The surveys were delivered using a signal‐contingent design on a quasi‐random schedule (random within four time windows within each day) between 10 am and 10 pm each day. The average response latency (time between receiving and answering a prompt) was 25 min and respondents could respond up to 2 h after a prompt was issued. Each survey was designed to take <2 min and included questions on current context, substance use, provocations, stress, aggression, and affect. The full questionnaire is provided in Supplementary Materials: https://osf.io/ez879.

Participants received an incentive for their participation, scaled to the level of response up to a maximum of 50CHF for onboarding and completing >70% of the surveys in both week 1 and week 2 of the study. Specifically, participants received 10CHF for downloading the application and setting up the study, 10CHF for achieving a response rate of at least 70% in week 1, and 20CHF for achieving a response rate of at least 70% in week 2. All participants were offered the same incentive structure. The study was implemented by the Decision Science Laboratory group at the Swiss Federal Institute for Technology (ETH), Zurich, Switzerland. Participants could contact an email helpline at any stage during the study if they encountered any difficulties.

### 2.3 Measures

#### 2.3.1 Outcome variables

Adherence was measured as the number of prompts responded to over the 2‐week measurement period, with a possible maximum of 56. This was measured based on the EMA component of the data collection.

#### 2.3.2 Predictor variables

Predictor variables were selected based on the a priori plausibility of an association with adherence based on past empirical research. They were measured in the age 20 main survey component of the z‐proso study. Given the exploratory nature of the study, several predictors, including broader psychosocial well‐being outcomes were included even if they had been previously little‐explored as predictors of research participation.

*Migration background* was based on a self‐reported migration background item administered at the age 15 wave of z‐proso coded dichotomously (1 = immigrant background; 2 = not of immigrant background). Of the participants included in this study, 36.9% were of immigrant background.

*SES* was estimated based on the maximum of maternal and paternal International Socio‐economic Index of Occupational Status (ISEI; Ganzeboom et al., 1992) reported youth at age 13/15. The ISEI system codes occupations, with higher scores representing more prestigious occupations. For example, an unskilled factory worker is assigned a score of 24, a plumber a score of 36, a social worker a score of 56, and a medical doctor 88.

*ADHD symptoms, depression, anxiety, psychosis, aggression,* and *prosociality* were measured at age 20 using an adapted version of the self‐reported *Social Behavior Questionnaire* (SBQ; Tremblay et al., 1991). It measures symptoms/behaviours on a 5‐point scale from 1 = *never* to 5 = *very often*. It includes 9 ADHD items (omega reliability = 0.87), 9 depression items (*ω* = 0.90), 4 anxiety items (*ω* = 0.70), 6 psychosis items (*ω* = 0.64), 19 aggression items (*ω* = 0.82), and 10 prosociality items (*ω* = 0.83). The psychosis items were not included in the original SBQ, but adapted from the Community Assessment of Psychic Experiences. Composite scores were derived for each sub‐scale by averaging the item scores. Previous analyses have supported the reliability, convergent validity, factorial validity, sensitivity to intervention effects, and longitudinal measurement invariance of the SBQ in the current sample (Murray, Booth, et al., 2019; Murray, Eisner, et al., 2017; Murray, Eisner, et al., 2019; Murray, Obsuth, et al., 2017).

*Substance use* was assessed at age 20 by providing a checklist of substances with the instruction: *‘Listed below are some drugs, intoxicants and other substances. Have you ever taken any of them and if yes, how many times in the last 12 months*?’. The current study examined the common forms of substance use, including, tobacco, alcohol (beer/wine/alcopops), alcohol (spirits), and cannabis use. Each substance use was measured using a single item on a six‐point scale: 1 = *never*, 2 = *once*, 3 = *2–5 times*, 4 = *6–12 times (monthly)*, 5 = *13–52 times (weekly)*, and 6 = *53–65 times (daily)*. These items were treated as continuous in the analyses given that they had >5 response options, all of which were utilised and they were only used as predictors, not outcomes in the regression models (see e.g., Rhemtulla et al., 2012 for a discussion of when categorical variables can be reasonably treated as continuous).

*Self‐injury* was measured at age 20 using a single question that asked how frequently participants had intentionally self‐injured in the previous month. Examples of self‐injurious behaviour include cutting an arm, tearing wounds open, hitting one's head, and tearing out one's hair. It assesses self‐injury on a 5‐point scale, from 1 = *never* to 5 = *very often*.

*Delinquency* was measured at age 20 using a variety index that is, the sum of the incidence of 7 delinquent acts in the previous 12 months: stealing at home, shoplifting goods worth less than 50CHF, shoplifting goods worth more than 50CHF, vehicle theft, fare dodging, vandalism, and assault. To form these scores, each delinquent act is scored dichotomously (0/1) and these dichotomous scores are averaged. Variety indices are considered advantageous over composite scores based on the sum of the frequency of delinquent acts (e.g., the sums of scores based on how often each act took place) because they avoid composite scores being disproportionately driven by frequent but minor offences (e.g., high scores due to regular fare dodging but in the absence of any serious delinquent acts).

*Psychopathy* was measured at age 20 using a 6‐item subscale of the *Short Dark Triad* scale (Jones & Paulhus, 2014). Items measured aspects of psychopathy such as callous affect, erratic lifestyle, antisocial behaviour, and short‐term manipulation (example item: ‘Payback needs to be quick and nasty’). Responses are recorded on a 4‐point scale from *false* to *true* and item scores are averaged to obtain a composite psychopathy score (*ω* = 0.73). Previous psychometric studies have supported the reliability and validity of these scores (Jones & Paulhus, 2014).

*Low self‐control* was measured at age 20 using an adapted 10‐item version of Grasmick's Low Self‐control scale (Longshore et al., 1996). It measures self‐control in terms of impulsivity, risk‐seeking, preference for physical over cognitive activity, temper, and self‐centredness. Responses are recorded on a 4‐point scale from 1 = *fully untrue* to 4 = *fully true* and averaged to provide a composite self‐control score (*ω* = 0.72). This scale and its variations have been widely used and evaluated in studies of crime and deviance. Previous studies in the current sample have supported the reliability, longitudinal invariance, factorial validity, and convergent validity of these scores (Murray et al., 2016).

*Moral shame* was measured at age 20 using a 3‐item version of the *Guilt and Shame* scale (Wikström & Butterworth, 2006), which captures an individual's tendency to feel guilt/shame when they know they have done something wrong. The items are measured on a 4‐point Likert‐type scale from 1 = *false* to 4 = *true* and averaged to provide a composite score (*ω* = 0.76).

*Self‐efficacy* was measured at age 20 using a 5‐item scale adapted from the 10‐item scale proposed by (Schwarzer & Jerusalem, 1999) with items such as ‘If there are difficulties, I find ways or means to overcome them’. Responses are recorded on a 4‐point Likert‐type scale from 1 = *false* to 4 = *true* and averaged to provide a composite self‐efficacy score (*ω* = 0.70).

*Social exclusion* was measured at age 20 using the *Perceived Social Exclusion Scale* (Bude & Lantermann, 2006). The version administered at the age 20 wave of z‐proso includes 6 items (e.g., ‘I feel like a stranger here’) with responses recorded in a 4‐point Likert‐type scale from 1 = *false* to 4 = *true*. Items are summed to provide a composite social exclusion score (*ω* = 0.88).

*Stress* was measured at age 20 using an abbreviated 4‐item version of the *Perceived Stress Scale* (Cohen, 1988). Responses are recorded on a 5‐point Likert‐type scale from 1 = *never* to 5 = *very often*. Scores were summed to provide a composite perceived stress score (*ω* = 0.85). The internal consistency reliability, factorial validity, and convergent validity of this scale has been supported in a previous study in the current sample (Murray, Xiao, et al., 2022).

*General trust* was measured at age 20 using a 3‐item measure adapted from the *World Values Survey* (example item: ‘most people can be trusted’) with responses recorded on a 4‐point Likert‐type scale from 1 = *true* to 4 = *false*. Item scores are averaged to obtain a composite general trust score (*ω* = 0.87).

### 2.4 Statistical procedure

First, we examined the unadjusted associations between each candidate predictor and adherence by regressing adherence on each of the predictors in separate models. We used full information maximum likelihood (FIML) estimation in lavaan (Rosseel, 2012). We used 95% bootstrapped confidence intervals with 1000 bootstrap samples to assess the significance of effects given the non‐normal distribution of the outcome variable (Rosseel, 2012; West et al., 1995). To gain further insights into the effects of these candidate predictors, we also fit a multi‐variable model entering all predictors simultaneously to provide mutually adjusted coefficients. This model was also fit in lavaan using FIML estimation and bootstrapped confidence intervals to assess statistical significance. Finally, to identify a more parsimonious model, we fit the multi‐variable model including a lasso penalty on the regression coefficients to aid in model selection within a regularised SEM framework (Jacobucci et al., 2016). We treated the penalty as a tuning parameter and selected a final model based on the model with the best Bayesian Information Criterion (BIC). As recommended, following fitting a regularised model, we re‐fit a model with the predictors with 0 coefficients in the regularised model removed but with no lasso penalty (Serang et al., 2017). For the regularised models it was necessary to standardise all predictors prior to model fitting.

### 2.5 Ethics and data sharing

This study was reviewed and approved by the University of Zurich's Faculty of Arts and Social Science's Ethics Committee. Written informed consent was obtained from participants prior to data collection. Data can be shared by reasonable request to the first or last author on signing a confidentiality agreement.

## 3 RESULTS

Descriptive statistics for the predictors are provided in Table 1. After removing participants who did not complete any prompts, the mean number of prompts completed by the *n* = 255 who remained in the analytic sample was 33.53 (*SD* = 15.87, range = 1–56; median = 39). The bivariate correlations between adherence and the predictors are provided in Supplementary Materials. Tetrachoric, polychoric, polyserial, and Pearson correlations were used to estimate these as relevant depending on the scale of the variables involved. These suggested a degree of collinearity between predictors. For example, there were gender differences in most predictors (except psychosis‐like symptoms, prosociality, low self‐control, self‐efficacy, and stress) and different mental health symptoms tended to be significantly correlated.

Results of the univariate regressions of adherence on the candidate predictors are summarised in Table 2. These unadjusted models suggested that individuals who were higher in aggression, higher in tobacco use, higher in psychopathy, higher in delinquency, and higher in low self‐control had lower adherence with the EMA protocol. Individuals who were higher in a tendency to feel guilt/shame when they perceived that they have done something wrong and those who were higher in self‐injury had higher adherence. None of migration background, SES, gender, self‐efficacy, ADHD symptoms, depression, anxiety, prosociality, general trust, psychotic‐like symptoms, stress, social exclusion, alcohol use (neither spirits nor beer/wine/alcopop consumption) nor cannabis use were significantly related to EMA adherence.

**TABLE 2:** Effects of respondent characteristics on adherence scores.

	*B*	SE	95% CI for *B*		Standardised *B*	Sig
			Lower	Upper		
Gender	2.331	2.02	−1.568	6.388	0.072	ns
Migration status	0.200	2.114	−4.06	4.325	0.006	ns
SES	0.021	0.05	−0.074	0.122	0.026	ns
ADHD symptoms	1.283	1.419	−1.537	4.082	0.054	ns
Depression symptoms	1.424	1.232	−1.109	3.698	0.073	ns
Anxiety symptoms	1.214	1.186	−1.239	3.384	0.068	ns
Psychosis symptoms	−1.661	2.38	−6.322	2.766	−0.049	ns
Aggression symptoms	−7.574	3.799	−15.538	−0.726	−0.142	*
Prosociality	0.316	1.949	−3.769	4.222	0.01	ns
Tobacco use	−1.087	0.524	−2.148	−0.084	−0.128	*
Alcohol: Beer, wine, alcopops	0.857	0.737	−0.557	2.415	0.072	ns
Alcohol: Spirits	−1.009	0.833	−2.706	0.632	−0.08	ns
Cannabis	−0.485	0.601	−1.681	0.768	−0.05	ns
Self‐injury	4.005	1.622	0.724	7.083	0.112	*
Delinquency	−18.559	8.222	−33.911	−2.11	−0.134	*
Psychopathy	−4.288	2.073	−8.626	0.052	−0.13	ns
Low self‐control	−6.075	2.519	−10.793	−1.084	−0.15	*
Moral shame	3.303	1.536	0.127	6.304	0.135	*
Self‐efficacy	−3.814	2.235	−8.263	0.467	−0.109	ns
Social exclusion	2.646	1.608	−0.593	5.733	0.093	ns
Stress	0.543	1.088	−1.644	2.847	0.031	ns
General trust	1.301	1.557	−1.725	4.461	0.055	ns

*Note*: * = significant at *α* = 0.05 based on 95% bootstrapped confidence interval. Reference category for gender = male; reference category for migrant status = not of immigrant background.

Abbreviations: ADHD, attention deficit hyperactivity disorder; ns, non‐significant; SES, socioeconomic status.

Results of the mutually adjusted models including all predictors simultaneously are provided in Table 3. None of the univariable effects remained significant; however, alcohol use (beer/alcopops) became a positive predictor of adherence, while alcohol use (spirits) became a negative predictor. Notably, the two alcohol use predictors were very highly correlated (*r* = 0.74, *p* < 0.001; see Table S1). Together, all predictors explained 25.4% of the variance in adherence.

**TABLE 3:** Multiple regression results.

	*B*	SE	95% CI for *B*		Standardised *B*	Sig
			Lower	Upper		
Gender	0.954	2.493	−4.039	5.881	0.027	ns
Migration status	4.154	2.599	−0.628	9.157	0.118	ns
SES	−0.022	0.065	−0.151	0.105	−0.025	ns
ADHD symptoms	2.576	2.062	−1.837	6.316	0.102	ns
Depression symptoms	0.602	2.261	−3.598	5.011	0.029	ns
Anxiety symptoms	−0.241	1.777	−3.694	3.55	−0.012	ns
Psychosis symptoms	−4.054	3.07	−9.957	1.905	−0.111	ns
Aggression symptoms	−8.751	4.927	−18.373	1.396	−0.153	ns
Prosociality	−0.734	2.204	−4.783	3.921	−0.023	ns
Tobacco use	−0.495	0.647	−1.805	0.741	−0.054	ns
Alcohol: Beer, wine, alcopops	3.976	1.11	1.82	6.111	0.312	*
Alcohol: Spirits	−3.11	1.199	−5.527	−0.815	−0.228	*
Cannabis	0.027	0.747	−1.459	1.424	0.003	ns
Self‐injury	3.738	2.233	−0.642	8.289	0.097	ns
Delinquency	−9.447	8.998	−27.324	9.176	−0.063	ns
Psychopathy	0.51	3.03	−5.627	6.627	0.014	ns
Low self‐control	−0.126	3.649	−7.088	7.795	−0.003	ns
Moral shame	1.753	1.888	−2.142	5.088	0.067	ns
Self‐efficacy	−3.203	2.685	−8.556	1.704	−0.085	ns
Social exclusion	2.669	2.488	−2.336	7.173	0.087	ns
Stress	−0.517	1.92	−4.265	3.282	−0.027	ns
General trust	1.725	1.968	−1.927	5.733	0.068	ns

*Note*: * = significant at *α* = 0.05 based on 95% bootstrapped confidence interval. Reference category for gender = male; reference category for migrant status = not of immigrant background.

Abbreviations: ADHD, attention deficit hyperactivity disorder; ns, non‐significant; SES, socioeconomic status.

As a final step, we implemented predictor selection using lasso. In the tuning stage, the regularised SEM model with all predictors was optimised in terms of BIC with a lambda value of 0.008. This led to several candidate predictors being indicated for de‐selection (based on regression coefficients driven to 0 by the lasso penalty): depression, anxiety, cannabis use, and self‐control. The model re‐fit with only the selected predictors is provided in Table 4. The same predictors remained significant as in the full multi‐predictor model, namely alcohol use (beer/wine/alcopops) and alcohol use (spirits) where again the former was related to better adherence and the latter to worse adherence. Together, the predictors in this more parsimonious model explained 25.3% of the variation in adherence.

**TABLE 4:** Multiple regression results after model selection using lasso.

	*B*	SE	95% CI for *B*		Standardised *B*	Significance
			Lower	Upper		
Gender	0.842	2.349	−3.63	5.509	0.024	ns
Migration status	4.203	2.386	−0.374	8.971	0.119	ns
SES	−0.022	0.062	−0.137	0.106	−0.025	ns
ADHD symptoms	2.583	1.848	−1.161	6.013	0.102	ns
Psychosis symptoms	−3.98	2.971	−9.531	1.963	−0.109	ns
Aggression symptoms	−8.774	4.726	−18.301	0.161	−0.153	ns
Prosociality	−0.716	2.243	−5.176	3.772	−0.022	ns
Tobacco use	−0.494	0.613	−1.692	0.703	−0.054	ns
Alcohol use: Beer, wine, alcopops	3.980	1.099	1.957	6.102	0.313	*
Alcohol use: Spirits	−3.087	1.222	−5.63	−0.795	−0.226	*
Self‐injury	3.801	2.17	−0.501	8.066	0.099	ns
Delinquency	−9.441	8.843	−27.219	7.767	−0.063	ns
Psychopathy	0.421	2.537	−4.344	5.874	0.012	ns
Moral shame	1.743	1.959	−2.136	5.438	0.066	ns
Self‐efficacy	−3.27	2.661	−8.326	1.648	−0.087	ns
Social exclusion	2.908	2.31	−1.968	7.213	0.095	ns
Stress	−0.367	1.761	−3.821	3.297	−0.019	ns
General trust	1.748	1.936	−2.067	5.304	0.069	ns

*Note*: * = significant at *α* = 0.05 based on 95% bootstrapped confidence interval. Reference category for gender = male; reference category for migrant status = not of immigrant background.

Abbreviations: ADHD, attention deficit hyperactivity disorder; ns, non‐significant; SES, socioeconomic status.

## 4 DISCUSSION

The purpose of the present study was to explore which respondent characteristics predict adherence in EMA studies. The goal was to illuminate the psychological mechanisms underpinning (non‐)adherence and to identify possible targets for improving adherence. Building on previous research that has largely focused on sociodemographic, limited domains of psychiatric symptoms, and substance use variables as predictors, we explored an expanded set of candidate predictors. These included a range of sociodemographic, mental health, and psychosocial wellbeing indicators. Lower self‐control, higher aggression, higher psychopathy, higher tobacco use, and higher delinquency were associated with lower adherence whereas higher levels of self‐injury and a higher tendency to feel moral guilt or shame were associated with higher adherence. Beyond this, mental health and neurodevelopmental symptoms (ADHD, anxiety, depression, and psychosis symptoms) and broader markers of psychosocial wellbeing (self‐efficacy, social exclusion, stress, general trust, prosociality) were not associated with adherence. In multi‐variable adjusted analyses, none of the univariable effects remained significant, suggesting that their effects on adherence are overlapping. However, positive effects of consumption of beer/wine/alcopops and negative effects of the use of spirits on adherence emerged.

The relations observed between aggression, moral shame, tobacco use, low self‐control, psychopathy, and delinquency and adherence suggest that traits related to self‐regulation and anti‐social behaviour/emotions may be particularly important for influencing EMA adherence. This is important because these same constructs are often studied using EMA paradigms to illuminate the everyday processes underlying and representing the manifestation of individual differences in these traits (Murray et al., 2021; Murray et al., 2020; Scott et al., 2017; Timmons et al., 2019). Our findings here suggest that missingness in these studies may be non‐random with respect to between‐individual differences in these characteristics. This may, therefore, result in parameter bias in studies of these constructs if not suitably compensated for using appropriate missingness models. In studies on these topics, it will be important to, for example, not impose strict adherence thresholds (i.e., removing participants with adherence below a given percentage). Those deleted from the study will not be random with respect to the study variables of interest such that this practice could induce bias in the between‐person parameters.

These findings also suggest that achieving adherence may be more challenging in populations scoring on average at more extreme levels on these traits (e.g., in forensic populations). As such, strategies to promote adherence for individuals high on these traits should be explored. Drawing on existing knowledge of effective strategies to promote adherence in survey studies (Murray & Xie, 2023; Teague et al., 2018), this may include investing in building relationships with participants and the community they are being sampled from, highlighting the interest/benefits of the study to the particular population being sampled, reducing barriers (e.g., making smartphone applications as easy as possible to download and use), providing greater reinforcement for participation (e.g., different schedules of or enhanced incentives), lowered burdens (e.g., fewer items per prompt or reduced study durations), offering alternative data collection modes (e.g., web and smartphone‐based), and flexibility of scheduling data collections (e.g., allowing different start times or time windows of data collection within the day). It may also be beneficial to screen for these traits in intake surveys to facilitate the delivery of tailored EMA protocols dependent on trait levels, that is, implementing ‘adaptive’ study designs that aim to maximise responses by tailoring data collection protocols to characteristics associated with a greater risk of non‐response (Lynn, 2017). However, this would need to be explored further in EMA contexts as it could also have a paradoxical effect of reinforcing the idea that data collection is difficult. Oversampling of participants with characteristics that put them at greater risk of low EMA adherence has also been proposed (Martinez et al., 2021). While this is not a method of improving adherence, it can mitigate the lower amount of data that may be gathered from sub‐groups with lower adherence. Finally, higher sampling frequencies coupled with an acknowledgement that some missed prompts are normal and expected could be explored as a means to increase feasibility and acceptability to participants.

However, more research will be required to establish effective strategies for improving adherence for ‘lower adherence’ individuals as the vast majority of previous research into promoting survey responses in groups at risk of low adherence has been in traditional survey designs. There are very few strategies that have been found to be effective for improving engagement in EMA studies and it is not yet clear how findings from traditional surveys might generalise to EMA designs in terms of specific strategies (Daniore et al., 2022; Murray & Xie, 2023). Further, it is important to acknowledge that not all respondent characteristics associated with adherence will respond to the same types of interventions to promote adherence. For example, it can be speculated that the links between antisocial traits and adherence we identified may reflect a lower *willingness* to adhere to an EMA protocol and, as such, interventions for sub‐populations high in these traits may focus on ways to design and frame a study to increase the extent to which high levels of engagement can be seen as beneficial to the respondent (e.g., based on an incentive offered). On the other hand, characteristics such as low self‐control may be associated with a lower *ability* to respond to prompts, and individuals with these traits may respond better to interventions that provide more support or reduced barriers (e.g., more reminders and encouragement, check‐ins with study staff, greater flexibility in data collection). To help with tailoring protocols to specific groups, task analysis could be used to anticipate the potential difficulties in adherence in a given population and protocols designed to address these (e.g., Adams et al., 2013). Further, co‐production approaches may be helpful for designing population‐specific strategies to promote adherence (Murray & Xie, 2023; Soyster & Fisher, 2019).

We also found a general lack of associations between mental health, neurodevelopmental symptoms, and wellbeing and adherence. Mental health EMA and ecological momentary intervention (EMI) studies are increasingly popular across a wide range of mental health issues (Balaskas et al., 2021); however, concerns have been raised about whether psychiatric populations may have additional difficulty with complying with EMA/EMI protocols. Our findings suggest that at the general population level, mental health symptoms are generally not associated with EMA adherence, therefore, the presence of mental health issues may not represent an important barrier to sustained engagement with smartphone‐based applications for symptom recording. Previous studies have been inconsistent in terms of the evidence for an effect of mental health issues on EMA adherence (Rintala et al., 2019; Vachon et al., 2019; van Genugten et al., 2020). Potentially some domains (e.g., mania) of mental health may be more relevant for EMA adherence than others and/or the effects are only seen in severe forms of mental illness; however, this requires further examination (Gershon et al., 2019; Hartley et al., 2014). Our study, on the other hand, did not include symptoms of ‘severe mental illness’ nor explicitly recruit clinically diagnosed patients. Future studies that take a more transdiagnostic approach and examine the effects of a wide range of mental health issues and severities on EMA adherence within the same study (to complement existing between‐study comparisons in reviews) will be valuable for providing some clarity on this question. Further, taking a more symptom‐level perspective may help reveal whether there are particular symptoms within mental health conditions that are more important than others for influencing adherence.

The lack of association with ADHD symptoms was also notable. There are a growing number of EMA studies in ADHD (Miguelez‐Fernandez et al., 2018) and EMIs are seen as promising for this patient group (Koch et al., 2021). However, there has been a lack of direct research into whether and how ADHD symptoms might impact adherence, despite concerns that ADHD symptoms (associated with e.g., forgetfulness, difficulties with organisation, waning motivation) might make EMA adherence more difficult. Consistent with our findings, a recent review suggested that adherence rates in EMA studies of ADHD populations are approximately comparable to those in general EMA studies (Koch et al., 2021; Wrzus & Neubauer, 2023). It is possible that despite potential difficulties in other domains (e.g., medication adherence) the particular features of EMA (e.g., automatic smartphone‐delivered reminders, minimal need to deviate from normal routines to achieve adherence) mean that it does not pose problems for individuals with ADHD symptoms.

There was one major exception to a lack of association between mental health and adherence in our study, namely, that higher levels of self‐injury were associated with *improved* adherence. There are several possible explanations for this. One comes from the functional model of self‐injury which suggests that, self‐injury may escalate through interpersonal positive reinforcement, in which the behaviour is followed by the occurrence or increase in a desired social event, such as attention or support (Nock, 2010). As such, those who self‐injure may be more likely to report events to gain attention/support, hence increasing adherence in the context of EMA designs which allow users to document their symptoms and behaviours. A second possibility may relate to the fact that those who self‐injure may experience higher levels of shame‐proneness (VanDerhei et al., 2014) and this may extend to anticipatory feelings of shame related to prompt non‐completion. The association between self‐injury and moral shame was, however, modest in the present study (*r* = 0.10), suggesting that this is likely to be a partial explanation at best. A third possibility may relate to the fact that self‐injury is related to perfectionism traits (Gyori & Balazs, 2022) which could also drive higher response rates to EMA surveys. Additional research is needed to determine whether the association between self‐injury and improved adherence replicates in other contexts and to explore the mechanisms involved.

In our analyses mutually adjusting for all predictors none of the effects observed in univariate analyses remained significant. This suggests that the effects of these variables were overlapping (indeed, this was supported by their bivariate inter‐correlations, see Table S1). However, when using lasso to facilitate the selection of only a subset of predictors to provide a more parsimonious model, the majority of variables were selected despite many of them having non‐significant coefficients. This model explained ∼25% of the variance in adherence, highlighting that although adherence is predictable from respondent characteristics, there are likely to be a large number of characteristics each of small effect that need to be measured to provide good estimates of adherence. As such, studies seeking to predict who is at greatest risk of adherence are unlikely to be able to rely on just one or two respondent characteristics and instead should expect to measure a relatively broad range of characteristics.

In these analyses we also found that alcohol use emerged as a significant predictor of adherence; however, the direction of the effect depended on the type of substance use. Use of beer/alcopops was positively associated with adherence, while use of spirits was negatively associated when adjusting for each other and all other predictors. It was notable that this latter finding only emerged when adjusting for (i.e., the effect was conditional on) beer/alcopop use. A possible explanation for this is that the use of spirits over and above beer/alcopops may reflect more problematic alcohol use. On the other hand, some alcohol use is quite normative among young adults (Willoughby et al., 2021) and abstaining from or low alcohol use could represent a marker of, for example, poor health or social functioning. Previous studies have suggested that general population variation in alcohol use (not distinguishing beer/alcopops vs. spirits) may not, in fact, be associated with EMA adherence (Howard & Lamb, 2022). In contrast, participants with a substance use disorder have been shown to have lower average EMA adherence (Jones et al., 2019). It has been speculated that this relates to more chaotic lifestyle habits that make EMA participation more challenging to sustain (Jones et al., 2019). This finding also points to the importance of differentiating different types and severities of substance use when examining predictors of adherence.

### 4.1 Limitations and future directions

It is important to highlight the limitations of the present study. First, it is important to note the boundaries on the generalisability of findings. For example, while the z‐proso cohort has been found to be largely representative of the underlying same‐aged population and the D2M sub‐study sample to differ only minimally from it on a range of variables, our sample is slightly selective with respect to variables such as SES and aggression (Eisner et al., 2018; Murray et al., 2020). It also includes a higher proportion of females than males, in line with the general trend for male gender to predict lower levels of research participation (Hawkes & Plewis, 2006; Watson & Wooden, 2009). The sample was also limited to emerging adults in Switzerland and our results may not generalise to other age groups and settings. For example, though EMA studies are particularly popular in adolescence and young adulthood, they are used across a diversity of age groups and it will be important to investigate predictors in other age groups. Similarly, to take account of trends in smartphone use, it will be valuable to update the evidence on EMA adherence predictors regularly to capture changes across cohorts of individuals living in different times. It will also be particularly important to replicate our findings in clinically ascertained samples in the future to evaluate whether the findings generalise to those with a psychiatric diagnosis.

A further limitation on generalisability concerns the EMA design. There are a large number of design parameters in EMA studies such as the frequency, pattern and length of the EMA surveys, the duration of the data collection, the use of a signal, event, or interval contingent design, the content of the measures, whether recruitment and on‐boarding are online or remote, and incentives (Bolger & Laurenceau, 2013). It is possible that these could interact with predictors of adherence (e.g., individuals with mental health issues may be more interested in a mental health EMA study or mental health may be less important for short duration, lower burden studies) and future studies will be needed to explore these effects. We were also not able to, for example, examine how incentives impacted adherence, whether they may change participants' behaviour (e.g., staying home or awake to not miss prompts), and whether incentives offered interacted with respondent characteristics since all respondents were offered the same incentive structure.

Second, our sample size was relatively large for an EMA study but modest in size overall. This, for example, limited our power to examine gender differences in participation predictors (in combination with the under‐representation of genders other than female). As EMA is becoming more feasible at scale, it would be valuable to replicate and extend our study to a larger sample and to examine more complex models including, for example, interactions of predictors (e.g., examining interactions between traits and gender).

Third, we did not have information on participants for prompts missed, that is, we did not have knowledge of their concurrent emotional states, cognitions, and experiences at these times. This means we were unable to examine concurrent predictors of prompt‐wise missingness and how respondent characteristics may interact with these to predict adherence. This also means that the specific implications of lower adherence for some groups for parameter bias is unclear. For example, it is not clear whether respondents with characteristics associated with lower adherence (e.g., those with higher aggression, delinquency) provided responses that were a random sample of the responses that they would have provided with higher adherence, or whether their responding was not only reduced relative to participants, but also selective. This would have more serious implications and could induce bias not only at the between‐person level, but also in within‐person parameters.

Finally, there are some limitations in our measures. Our measure of SES was based on the age 13 and 15 waves of z‐proso; however, by age 20, young people's SES may be less dependent on the occupations of their parents. For other measures, the z‐proso study used abbreviated versions of established measures, reflecting the space constraints within comprehensive large‐scale surveys. Almost all multi‐item measures showed internal consistency reliability values (McDonald's omega) >0.70; however, it fell short of this for the psychosis symptoms measure. The most likely impact of this is to attenuate the association of its scores with adherence. Our measure of adherence also assumed that respondents were answering honestly and with sufficient effort required to give accurate answers; however, careless responding is a known issue in EMA (Jaso et al., 2021). We did not distinguish between genuine and careless responding in our measure of adherence because our primary interest was in whether participants responded to a prompt at all or not (doing so can provide some information, such as the automatic recording of GPS co‐ordinates in our study). However, defining non‐adherence to include careless responding (to the extent that it is possible to detect) may reveal different patterns of predictors.

An important future direction will be to develop and experimentally test different regimes for improving adherence in EMA. These could be informed by approaches such as applied behaviour analysis, which provide some strategies for increasing establishing and maintaining desired behaviours. The long‐term repetitive data collection characteristic of many EMA protocols requires participants to ‘wait’ for the delayed rewards (incentives) and relies on future expectation, self‐control, and delay gratification (Daniel et al., 2015). As such, strategies that deliver immediate compensation after each prompt or that provide alternative reinforcers (e.g., a token or sticker) that can be accumulated to earn a final more valuable incentive may be helpful. An incentive hierarchy could be employed whereby both each data collection and the accumulation of adherence with multiple data collections are rewarded (Ivy et al., 2017).

A second future direction concerns the possibility of using EMA adherence data to predict mental health or behavioural outcomes. While we focused here on the prediction of low adherence, our results equally suggest that lower adherence could be informative about respondent characteristics. An interesting extension of this is whether changes in adherence could predict important changes in participants' habits, such as the onset of a period of problematic alcohol use. There is some preliminary evidence that missingness in EMA may be associated with negative and positive affect outcomes (Cursio et al., 2019; Sokolovsky et al., 2014), for example, that stress or low mood might precede a missed prompt (Murray et al., 2023); however, there has been little exploration of the extent to which adherence in EMA predicts later outcomes.

## 5 CONCLUSIONS

Lower self‐control and a lack of moral guilt/shame, higher psychopathy, and higher delinquency, psychopathy, aggression, and tobacco use were related to higher EMA non‐adherence. Tailored EMA strategies may be beneficial for improving adherence for individuals scoring high on these characteristics.

## AUTHOR CONTRIBUTIONS

**Aja Murray**: Conceptualization; formal analysis; funding acquisition; writing—original draft preparation (lead). **Yi Yang**: Writing—review and editing; writing—original draft preparation (supporting). **Xinxin Zhu**: Writing—review and editing; writing—original draft preparation (supporting). **Lydia Speyer**: Writing—review and editing. **Ruth Brown**: Writing—review and editing. **Manuel Eisner**: Writing—review and editing; funding acquisition; project administration. **Denis Ribeaud**: Writing—review and editing; funding acquisition.

## CONFLICT OF INTEREST STATEMENT

All other authors declare that they have no conflicts of interest.

## Supporting information

Table S1

## Data Availability

The data that support the findings of this study are available on request from the corresponding author. The data are not publicly available due to privacy or ethical restrictions.
