# Supplementary material for: Respondent characteristics associated with adherence in a general population ecological momentary assessment study
Source: Int J Methods Psychiatr Res. 2023 May 15;32(4):e1972. doi: 10.1002/mpr.1972 (PMC10698810; doi:10.1002/mpr.1972)
Supplement: Supplementary file 1 — Table S1 [file MPR-32-e1972-s001.docx]

**Supplementary Materials**

**Table S1: Bivariate correlations between compliance and predictors**

|  | | **1** | | **2** | | **3** | | **4** | | **5** | | **6** | | **7** | | **8** | | **9** | | **10** | | **11** | | **12** | | **13** | | **14** | | **15** | | **16** | | **17** | | **18** | | **19** | | **20** | | **21** | | **22** | | **23** | |
| --- | --- | --- | --- | --- | --- | --- | --- | --- | --- | --- | --- | --- | --- | --- | --- | --- | --- | --- | --- | --- | --- | --- | --- | --- | --- | --- | --- | --- | --- | --- | --- | --- | --- | --- | --- | --- | --- | --- | --- | --- | --- | --- | --- | --- | --- | --- | --- |
| 1. Compliance | | - | | <.001 | | <.001 | | <.001 | | <.001 | | <.001 | | <.001 | | <.001 | | <.001 | | .012 | | <.001 | | <.001 | | <.001 | | <.001 | | <.001 | | <.001 | | .001 | | .008 | | <.001 | | .007 | | <.001 | | .012 | | <.001 | |
| 2. Gender | | .11 | | - | | - | | .003 | | .005 | | <.001 | | .027 | | .114 | | .029 | | .829 | | <.001 | | <.001 | | <.001 | | <.001 | | <.001 | | <.001 | | .002 | | .227 | | <.001 | | .299 | | .003 | | .416 | | <.001 | |
| 3. Migration | | .02 | | -.02 | | - | | <.001 | | .034 | | .002 | | .062 | | .032 | | .001 | | .674 | | <.001 | | <.001 | | <.001 | | <.001 | | <.001 | | <.001 | | .078 | | .254 | | <.001 | | .486 | | .003 | | .892 | | <.001 | |
| 4. SES | | .01 | | -.11 | | -.48 | | - | | .006 | | <.001 | | .006 | | .002 | | <.001 | | .009 | | <.001 | | <.001 | | <.001 | | <.001 | | <.001 | | <.001 | | .004 | | .044 | | <.001 | | .021 | | <.001 | | .084 | | <.001 | |
| 5. ADHD | | .07 | | .11 | | .01 | | .05 | | - | | <.001 | | .010 | | .051 | | .022 | | .137 | | <.001 | | <.001 | | <.001 | | <.001 | | <.001 | | <.001 | | .033 | | .090 | | .002 | | .220 | | <.001 | | .025 | | <.001 | |
| 6. Depression | | .07 | | .26 | | .06 | | -.01 | | .61 | | - | | .004 | | .005 | | .001 | | .010 | | <.001 | | <.001 | | <.001 | | <.001 | | <.001 | | <.001 | | .001 | | .003 | | <.001 | | .003 | | .001 | | <.001 | | <.001 | |
| 7. Anxiety | | .08 | | .55 | | .09 | | -.12 | | .57 | | .74 | | - | | .028 | | .005 | | .294 | | <.001 | | <.001 | | <.001 | | <.001 | | <.001 | | <.001 | | .035 | | .045 | | <.001 | | .029 | | .007 | | .355 | | <.001 | |
| 8. Psychosis | | -.03 | | .10 | | .08 | | -.06 | | .43 | | .57 | | .51 | | - | | .008 | | .341 | | <.001 | | <.001 | | <.001 | | <.001 | | <.001 | | <.001 | | .086 | | .264 | | <.001 | | .151 | | .003 | | .553 | | <.001 | |
| 9. Aggression | | -.14 | | .02 | | .28 | | -.10 | | .51 | | .40 | | .35 | | .28 | | - | | .361 | | <.001 | | <.001 | | <.001 | | <.001 | | <.001 | | <.001 | | .107 | | .131 | | <.001 | | .111 | | .001 | | .012 | | <.001 | |
| 1. Prosociality | | .00 | | .19 | | -.01 | | .06 | | .15 | | .10 | | .17 | | .07 | | .12 | | - | | .009 | | <.001 | | .007 | | <.001 | | <.001 | | <.001 | | .158 | | .441 | | <.001 | | .863 | | .016 | | .743 | | <.001 | |
| 11. Tobacco | | -.13 | | .09 | | -.04 | | -.01 | | .10 | | .03 | | .06 | | .09 | | .25 | | .10 | | - | | <.001 | | <.001 | | <.001 | | <.001 | | <.001 | | <.001 | | <.001 | | <.001 | | .001 | | <.001 | | .002 | | <.001 | |
| 12. Beer/wine | | .10 | | -.21 | | -.39 | | .39 | | .08 | | -.04 | | -.12 | | -.05 | | .02 | | .07 | | .24 | | - | | <.001 | | <.001 | | <.001 | | <.001 | | <.001 | | <.001 | | <.001 | | <.001 | | <.001 | | <.001 | | <.001 | |
| 13. Spirits | | -.08 | | -.22 | | -.24 | | .27 | | .04 | | -.01 | | -.14 | | -.03 | | .09 | | .03 | | .33 | | .74 | | - | | <.001 | | <.001 | | <.001 | | .001 | | <.001 | | <.001 | | .001 | | <.001 | | .001 | | <.001 | |
| 14. Cannabis | | -.03 | | -.14 | | -.17 | | .13 | | .19 | | .10 | | .04 | | .15 | | .14 | | .01 | | .43 | | .36 | | .37 | | - | | <.001 | | <.001 | | <.001 | | <.001 | | <.001 | | <.001 | | <.001 | | <.001 | | <.001 | |
| 15. Self-injury | | .11 | | .49 | | -.25 | | .05 | | .26 | | .47 | | .46 | | .31 | | .13 | | .11 | | .04 | | -.05 | | -.03 | | .13 | | - | | <.001 | | <.001 | | <.001 | | <.001 | | <.001 | | <.001 | | <.001 | | <.001 | |
| 16. Delinquency | | -.13 | | -.19 | | .02 | | .04 | | .25 | | .12 | | .04 | | .20 | | .38 | | .00 | | .23 | | .19 | | .23 | | .33 | | -.01 | | - | | <.001 | | <.001 | | <.001 | | <.001 | | <.001 | | <.001 | | <.001 | |
| 17. Psychopathy | | -.12 | | -.20 | | .22 | | -.09 | | .28 | | .20 | | .15 | | .18 | | .57 | | -.12 | | .23 | | .07 | | .19 | | .27 | | .06 | | .37 | | - | | .089 | | .001 | | .174 | | .018 | | .223 | | <.001 | |
| 18. Low SC | | -.15 | | -.11 | | .00 | | -.01 | | .25 | | .07 | | .01 | | .13 | | .47 | | -.09 | | .27 | | .12 | | .25 | | .32 | | .00 | | .37 | | .63 | | - | | .001 | | .660 | | .016 | | .775 | | <.001 | |
| 19. Shame | | .13 | | .37 | | -.16 | | -.04 | | .05 | | .06 | | .17 | | .01 | | -.15 | | .33 | | -.05 | | -.06 | | -.14 | | -.17 | | .10 | | -.36 | | -.39 | | -.34 | | - | | .006 | | <.001 | | .002 | | <.001 | |
| 2. Self-efficacy | | -.14 | | -.17 | | -.09 | | .05 | | -.33 | | -.44 | | -.35 | | -.23 | | -.17 | | .11 | | .12 | | .12 | | .04 | | .01 | | -.28 | | -.05 | | .03 | | .14 | | -.09 | | - | | .001 | | .642 | | <.001 | |
| 21. Social excl. | | .10 | | .17 | | .00 | | .00 | | .52 | | .73 | | .59 | | .47 | | .29 | | .02 | | .00 | | -.06 | | -.03 | | .14 | | .47 | | .15 | | .24 | | .11 | | .03 | | -.40 | | - | | .005 | | <.001 | |
| 22. Stress | | .04 | | .33 | | .02 | | .07 | | .65 | | .75 | | .70 | | .47 | | .44 | | .11 | | .05 | | -.04 | | -.05 | | .10 | | .32 | | .12 | | .22 | | .11 | | .08 | | -.42 | | .56 | | - | | <.001 | |
| 23. Trust | | .04 | | -.14 | | -.30 | | .26 | | -.26 | | -.29 | | -.28 | | -.17 | | -.25 | | .01 | | -.13 | | .10 | | -.03 | | -.09 | | -.22 | | -.13 | | -.21 | | -.09 | | .04 | | .28 | | -.32 | | -.33 | | - | |
|  | |  | |  | |  | |  | |  | |  | |  | |  | |  | |  | |  | |  | |  | |  | |  | |  | |  | |  | |  | |  | |  | |  | |  | |

*Note.* Low SC= Low self-control; Social excl.= Social exclusion. Correlations below the diagonal, p-values above
